# A Diabetes Self-Management Program Designed for Urban American Indians

**Published:** 2009-09-15

**Authors:** Sarah Castro, Mary O'Toole, Carol Brownson, Kimberly Plessel, Laura Schauben

**Affiliations:** Transtria LLC. At the time of this study, Ms Castro was affiliated with Saint Louis University, Saint Louis, Missouri; Washington University, Saint Louis, Missouri; Washington University, Saint Louis, Missouri; Minneapolis American Indian Center, Minneapolis, Minnesota; Wilder Research, Minneapolis, Minnesota

## Abstract

**Background:**

Although the American Indian population has a disproportionately high rate of type 2 diabetes, little has been written about culturally sensitive self-management programs in this population.

**Context:**

Community and clinic partners worked together to identify barriers to diabetes self-management and to provide activities and services as part of a holistic approach to diabetes self-management, called the Full Circle Diabetes Program.

**Methods:**

The program activities and services addressed 4 components of holistic health: body, spirit, mind, and emotion. Seven types of activities or services were available to help participants improve diabetes self-management; these included exercise classes, educational classes, and talking circles.

**Consequences:**

Ninety-eight percent of program enrollees participated in at least 1 activity, and two-thirds participated in 2 or more activities. Program participation resulted in a significant improvement in knowledge of resources for managing diabetes.

**Interpretation:**

The Full Circle Diabetes Program developed and implemented culturally relevant resources and supports for diabetes self-management in an American Indian population. Lessons learned included that a holistic approach to diabetes self-management, community participation, and stakeholder partnerships are needed for a successful program.

## Background

Self-management behaviors are key to managing type 2 diabetes and achieving successful health outcomes. The American Diabetes Association identifies self-management as the cornerstone of care for diabetes (1), and the Centers for Disease Control and Prevention state that self-management education is a critical part of medical care for diabetes (2). Self-management behaviors include eating healthfully, being physically active, monitoring blood glucose, taking medications, solving problems, healthy coping, and reducing risks (3). Self-management increases healthy behaviors (4) and improves clinical outcomes (4-7).

The American Indian population has a disproportionately high rate of type 2 diabetes. Approximately twice as many American Indian and Alaska Native adults have diabetes as do non-Hispanic whites, and the problem is increasing. The prevalence of diabetes in young adult (aged 20-29 years) American Indians and Alaska Natives increased by 58% from 1990 through 1998, compared with a 9% increase in the US population as a whole (8). Because diabetes develops in American Indians at an earlier age, they live with diabetes longer and are, therefore, at higher risk for diabetes-related complications. American Indians are 3.5 times more likely than the general population to have kidney disease (8) and 3.5 times more likely to require lower-limb amputations (9). American Indians are also more likely to be physically inactive and have an unhealthy diet (10,11). These behaviors are associated with worse self-reported health status, even after controlling for socioeconomic status (12).

Little has been written about culturally appropriate self-management interventions in the American Indian population. Two small, well-controlled studies showed that culturally relevant lifestyle interventions can improve diabetes-related behaviors and clinical outcomes (13,14). Missing from these studies were formal, sustained relationships with American Indian community members, studies that addressed more than a small subsample of the American Indian population, and intervention activities that could be implemented in uncontrolled community settings. We describe the Full Circle Diabetes Program, which focused on building physical, spiritual, mental, and emotional supports for diabetes self-management, and discuss implications for others interested in improving resources for diabetes self-management among urban American Indians.

## Context

In 2003, the Robert Wood Johnson Foundation's Diabetes Initiative awarded the Minneapolis American Indian Center (MAIC) a Building Community Supports for Diabetes Care grant. MAIC, a community center that provides education and social services to American Indians, partnered with the Native American Community Clinic (NACC), a primary care clinic, and Wilder Research, the research and evaluation division of the Amherst H. Wilder Foundation, to develop and implement a comprehensive program that was relevant to the community, culturally appropriate, and provided resources to improve diabetes self-management. Both MAIC and NACC are in the Phillips neighborhood of south Minneapolis, and their services extend to the entire metropolitan area. Approximately 85% of clients served by both are American Indians from different tribes.

Beginning in February 2003, a program coordinator from MAIC facilitated communication among program partners and recruited community members to form a Diabetes Community Council. The program coordinator was not American Indian but spent time and effort building trust with community members. She attended community events, engaged in dialogue with American Indian elders, and repeatedly demonstrated her interest in their welfare. The Diabetes Community Council comprised American Indian community members and elders from the Minneapolis/Saint Paul area, many of whom had type 2 diabetes. The council represented the community and advised MAIC, NACC, and Wilder Research on program planning, development, and evaluation.

MAIC called their holistic approach to program development a "circle model" because of its close relationship to American Indian culture and values. Circles are ancient symbols of infinity, unity, and wholeness. Their circle model recognizes that all people contribute uniquely to the survival and vitality of a community. Therefore, leadership is shared; each person contributes according to his knowledge and abilities.

Using this approach, council members identified barriers to self-management by sharing personal stories; these barriers included infrequent blood glucose testing, poor eating habits, poor physical activity habits, difficulty coping with diabetes-related stress, and lack of knowledge of self-management resources. After discussing the gaps in available and accessible resources, the council worked with the program coordinator to develop activities and services to complement and expand existing resources. Before the program, no diabetes services coordinated between MAIC and NACC were offered to community members. Two key medical staff from NACC attended all the council meetings and consulted on medical issues as needed. In addition to its role in developing and coordinating program activities, NACC provided clinical services and monitored clinical measures of the participants. Together, the Diabetes Community Council, MAIC, NACC, and Wilder Research created the Full Circle Diabetes Program.

## Methods

From July 2004 through February 2007, a total of 255 adults with type 2 diabetes participated in the Full Circle Diabetes Program. Participants were recruited at MAIC and NACC; all clients, regardless of ethnicity, were offered the opportunity to enroll in the program. Participants completed an intake form to document their characteristics at program entry. In addition, they completed a lifestyle survey to assess knowledge of resources at program entry, annually, and at program exit (15). Activities of the Full Circle Diabetes Program encompassed 4 dimensions: body, spirit, mind, and emotion. Program activities were developed for each dimension with the needs of the community in mind. Participants were not expected to participate in all program activities but were encouraged to participate in those that were most relevant to their self-management needs. Participation in program activities and services was tracked. The McNemar Test (2-sided) was used to test for change in knowledge of resources. Wilder Research provided MAIC and NACC with semiannual evaluation reports.

Both NACC and MAIC provided activities designed to improve physical health (body dimension). Diabetes-related clinical indicators were monitored at quarterly clinic visits at NACC to guide clinical management (data not reported); at these visits, participants received routine physical examinations, dietary advice, and screening for depression. MAIC and NACC jointly employed a diabetes case manager to contact patients, assess their needs, connect them with appropriate resources, and provide follow-up and support. The case manager also assisted with referrals to low-cost or free health care services and helped participants correctly fill out and file insurance forms.

During the first year of the program, exercise classes were offered 4 times per week at MAIC. As the program evolved, other exercise activities, such as water aerobics, walking, stretching, and light weight lifting, were also offered at MAIC and through partnerships with local fitness facilities. Free gym memberships, physical therapy services, walking groups, and consultations for home exercise programs were among the options offered. In response to a request from participants and the Diabetes Community Council, nutritional consultations were added to assess participants' diets and develop healthier meal plans that were realistic for participants with budget constraints.

The spiritual dimension of holistic health was addressed by honoring American Indian culture and fostering a sense of belonging to something larger than oneself. For example, NACC clinicians were comfortable with supporting traditional healers in providing alternative health care for participants who were interested in or already receiving treatment from these healers. The Diabetes Community Council honored tradition by offering a blessing before every council meeting. The meetings encouraged participants to share personal testimonies, which helped them feel less alone in their challenges and motivated them to make healthful life changes. Intergenerational events, such as diabetes health fairs, invited everyone in the community to participate, regardless of age. These events helped to bring all generations together to learn, have fun, and celebrate culture. Caregiver ceremonies acknowledged family, friends, and health professionals for supporting diabetes health.

The Full Circle Diabetes Program offered several opportunities to improve the mind dimension by increasing participant knowledge about diabetes and self-management. BASICS curriculum education classes, developed by the International Diabetes Center, focused on 6 topic areas: introduction to diabetes, nutrition, managing diabetes, health for a lifetime (full body health), physical activity, and stress management (16). This 5-class curriculum was offered through monthly BASICS dinners during the first year of the program. During the remaining year and a half, two 5-week workshops were offered in place of the monthly dinners, so that newly diagnosed participants or those with poorly controlled diabetes could move through the curriculum more quickly. In addition, monthly diabetes breakfasts gave participants the opportunity to share a healthy meal and benefit from ongoing diabetes education.

As the program evolved, the Stanford University Chronic Disease Self-Management Program (renamed Living in Balance) was added as another educational component (17). The program helped participants learn skills and set goals to better manage their diabetes and any other chronic conditions. To help sustain the program, leaders' training was offered at the beginning of year 2 to train program graduates to become peer teachers. To be eligible for the leaders' training, participants had to have their diabetes under control and have strong communication and listening skills.

Emotional support activities were included in the Full Circle Diabetes Program to address the emotion dimension and combat depression. For example, talking circles were informal group gatherings in which participants were encouraged to share concerns related to diabetes and other topics (18). The talking circles provided a safe place for people to ask for and receive support from their peers and to know that others shared their challenges. Lists of mental health resources were available at each talking circle, and a mental health counselor was available at NACC to assist with mental health services. Self-esteem was nurtured by engaging participants in planned outreach activities, such as presentations at local schools. As they gained skills and had positive experiences planning and executing these activities, participants developed the confidence necessary to become advocates for self-management.

Addressing each of the 4 dimensions of holistic health in a culturally sensitive way was the key to the Full Circle Diabetes Program. Although activities and services were categorized under a particular dimension, considerable overlap occurred. For example, services such as case management and nutritional consultations addressed primarily physical health (body) but also included educational elements (mind) and emotional support.

Grant funding ended for the Full Circle Diabetes Program in February 2007; however, many of the systems changes put in place during the program were maintained. The Diabetes Community Council continues to be active in the community but has changed its name to Community Health Council and has expanded its reach to include all health issues. Case management and nutrition counseling at NACC continues to provide guidance and support to diabetes patients to improve their self-management.

## Consequences

Entry data are available for 249 of the 255 people enrolled in the Full Circle Diabetes Program. Most participants were middle-aged women of American Indian or Alaska Native descent who had at least a high school education (Table 1). The average time since diagnosis of diabetes was 9 years, and comorbid conditions were common. Although most participants had health insurance at some time during the program, many did not have consistent, uninterrupted insurance throughout the program. Most reported having a regular personal physician and good support from family, friends, and the community. However, less than half knew how to access self-management resources.

Most participants reported taking their medicine as recommended most or all of the time, but only half tested their blood glucose 1 or more times per day, and most were not following a diabetes meal plan (Table 2). Although participation in some physical activity was common, less than one-third met physical activity standards (19).

At program entry, each participant was enrolled in case management. Participation in other program activities and services was variable (Table 3); however, 98% of program enrollees participated at least once in 1 activity or service, and two-thirds participated in 2 or more.

Diabetes breakfasts and nutritional consultations were the most popular activities. Seventy-five participants attended BASICS educational sessions, but on average each attendee completed only half of the lessons. In an effort to make the BASICS classes more accessible, dinner sessions were changed to workshops, but the workshops had less reach than the dinner sessions. Although only 16% participated in the MAIC exercise classes, an additional, unknown number participated in 1 or more of the exercise options that were added as the program evolved. Because these were held off-site, attendance records are not available. Attendance at each of the 10 intergenerational events ranged from 8 to 200 people and included both program participants and other community members. Program participants and other community members participated in talking circles. Although the data do not show which participants attended each activity, all dimensions of holistic health attracted participants.

Two-thirds of participants reported that information they learned in the Full Circle Diabetes Program helped them manage their diabetes. Program participation of any kind resulted in a significant improvement in knowledge of resources for managing diabetes (81 matched pairs, *P* = .04). Additionally, 98% of respondents reported that as a result of attending Living in Balance classes, they had made changes in 1 or more of the following behaviors: exercising, coping with diabetes stress, communicating with their health care provider, and improving their eating plan.

## Interpretation

The Full Circle Diabetes Program demonstrates that resources and supports for diabetes self-management can be developed, implemented, and successful in an American Indian population. Physical, spiritual, mental, and emotional supports for diabetes self-management were provided through a partnership among a community organization (MAIC), a health care clinic (NACC), and community members. Community members not formally enrolled in the program and from the broader metropolitan area participated in many program activities, which suggests that the Diabetes Community Council's goal of offering programs that fit American Indian culture was achieved. The Full Circle Diabetes Program developed successful partnerships and incorporated culture into all aspects of the program.

The experience of the Full Circle Diabetes Program offers several lessons for diabetes educators and organizations interested in implementing a comprehensive, community-supported diabetes self-management program for urban American Indians. The first lesson is that American Indian culture supports the use of a holistic approach to diabetes self-management because it emphasizes balance and harmony. The Full Circle Diabetes Program built on these cultural beliefs by using a circle model and offering activities in the 4 dimensions of holistic health to allow participants to choose options that best addressed their diabetes self-management needs. Previous studies have reported distinct self-management support preferences among subgroups of participants and suggest that a range of culturally appropriate supportive strategies should be offered (20,21).

The second lesson is that community participation is critical. The input of the Diabetes Community Council was essential to the success of the Full Circle Diabetes Program, a finding that is consistent with the expectations of community-based participatory approaches (22,23). The council had a vision for how diabetes self-management could be improved in their community. They helped design culturally appropriate community-based activities, became leaders for the Living in Balance classes, led talking circles, suggested intergenerational events, and participated in numerous outreach activities. The emphasis on community participation led to a self-management program that reflected and addressed the needs of the community.

The third lesson is that partnerships provide an opportunity to develop diabetes self-management programs and services. The Full Circle Diabetes Program's 4 main partners — MAIC, NACC, the Diabetes Community Council, and Wilder Research — brought together a clinic, community organizations, American Indian elders, spiritual leaders, community members, and an evaluation team. By combining expertise from each, the program offered a range of complementary program activities and services, engaged community members in diabetes self-management, and evaluated the program's effect. These partnerships were successful because each partner helped create the program, committed resources to it, and developed systems and procedures for working together to improve diabetes care. Participants entered the program from both the community and clinic because of this system. Sharing leadership resulted in more trust and strengthened relationships among the program partners because all voices were heard. Priorities and concerns of each partner were presented at council meetings, and solutions were considered collectively. The need to build partnerships that bring together complementary skills and resources is supported by studies that suggest clinic-community partnerships play a critical role in diabetes care and self-management (24).

Future work is needed to document that the Full Circle Diabetes Program can improve self-management behaviors among urban Native American Indians. More information, including a resource toolkit for developing a comprehensive diabetes self-management program for urban Native Americans, can be found at www.diabetesinitiative.org/programs/DIMAIC.html.

## Figures and Tables

**Table 1:** Characteristics at Program Entry of 249 Participants in the Full Circle Diabetes Program, Minneapolis, Minnesota, July 2004 through February 2007

**Characteristic**	% of Participants^a^
**Sex**	
Female	67
Male	32
**Age, y**	
<40	20
40-59	56
≥60	24
**Race/ethnicity**	
American Indian/Alaska Native	88
White	8
African American	5
Other	5
**Education, y**	
<12	32
≥12	64
**No. of years since diabetes diagnosis**	
≤2	27
3-9	24
≥10	42
**Clinical comorbidities**	
Hypertension	60
Dyslipidemia	48
Depression	31
**Access to care**	
Have health insurance	77
Have regular personal doctor	76
Receive regular medical care for diabetes	84
**Support from family, friends, community**	
Not at all	8
Rarely or sometimes	25
Most of the time or always	60
**Know how to access self-management resources**	
Not well at all	11
Not well or somewhat well	46
Well or very well	43

a Percentages may not total 100 because of nonresponse or multiple responses.

**Table 2:** Diabetes Self-Management Behavior at Program Entry of 249 Participants in the Full Circle Diabetes Program, Minneapolis, Minnesota, July 2004 through February 2007

**Behavior**	% of Participants^a^
**Tests blood glucose**	
Not at all	29
Less than once per day	14
Once per day or more	53
**Takes medication as recommended**	
Not at all	12
Rarely or sometimes	7
Most of the time or always	74
**Follows diabetes food plan**	39
**Participates in physical activity**	65
**Copes well with diabetes-related stress**	41
**Does not smoke cigarettes**	56

a Percentages may not total 100 because of nonresponse.

**Table 3:** Participation in Full Circle Diabetes Program Activities and Services, Minneapolis, Minnesota, July 2004 through February 2007

**Activity/Service**	% Who Attended at Least Once (N = 255)
Exercise classes^a^	16
Nutritional consultations	43
Intergenerational events	22
BASICS educational classes	30
Diabetes breakfasts	44
Living in Balance classes	11
Talking circles	11

a Minneapolis American Indian Center exercise classes only. Other exercise programs were added as the program evolved, but data on participation in these programs are not available.
